# Screening for *VPS35* mutations in Parkinson's disease

**DOI:** 10.1016/j.neurobiolaging.2011.10.032

**Published:** 2012-04

**Authors:** Una-Marie Sheerin, Gavin Charlesworth, Jose Bras, Rita Guerreiro, Kailash Bhatia, Thomas Foltynie, Patricia Limousin, Laura Silveira-Moriyama, Andrew Lees, Nicholas Wood

**Affiliations:** a Department of Molecular Neuroscience, UCL Institute of Neurology, Queen Square, London, UK; b Department of Motor Neuroscience, UCL Institute of Neurology, Queen Square, London, UK; c Sobell Department, Unit of Functional Neurosurgery, UCL Institute of Neurology, Queen Square, London, UK; d Reta Lila Weston Trust for Medical Research, UCL Institute of Neurology, Queen Square, London, UK; e Queen Square Brain Bank for Neurological Disorders, UCL Institute of Neurology, Queen Square, London, UK; f UCL Genetics Institute, University College London, London, UK

**Keywords:** Parkinson's disease, Genetics, VPS35, Population screening

## Abstract

Recently 2 groups have independently identified a mutation in the gene ‘vacuolar protein sorting 35 homolog’ (*VPS35* c.1858G>A; p.Asp620Asn) as a possible cause of autosomal dominant Parkinson's disease (PD). In order to assess the frequency of the reported mutation and to search for other possible disease-causing variants in this gene, we sequenced all 17 exons of *VPS35* in 96 familial PD cases, and exon 15 (in which the reported mutation is found) in an additional 64 familial PD cases, 175 young-onset PD cases, and 262 sporadic, neuropathologically confirmed PD cases. We identified 1 individual with the p.Asp620Asn mutation and an autosomal dominant family history of PD. Subsequent follow-up of the family confirmed an affected sibling and cousin who also carried the same mutation. No other potentially disease-causing mutations were identified. We conclude that the *VPS35* c.1858G>A mutation is an uncommon cause of familial Parkinson's disease in our population.

## 1 Introduction

Parkinson's disease (PD) is a common neurodegenerative disorder, affecting 2% of those over the age of 75 years (Mayeux et al., 1995). Neuropathologically, it is characterized by a progressive loss of dopaminergic neurons of the substantia nigra, associated with the formation of fibrillar aggregates composed of α-synuclein and other proteins (Lewy bodies and Lewy neurites) (Braak et al., 2003). Bradykinesia, rest tremor, rigidity, and postural instability represent the core clinical features (Poewe and Mahlknecht, 2009).

Although generally considered a sporadic disease, Mendelian forms of the disease are described and recent genome-wide association studies have shed considerable light on the genetic contribution to sporadic disease (International Parkinson Disease Genomics Consortium, 2011a; International Parkinson's Disease Genomics Consortium (IPDGC) and Wellcome Trust Case Control Consortium 2 [WTCCC2], 2011b). Robust evidence exists for 2 genes causing autosomal dominant PD (*SNCA*, *LRRK2*) and 3 genes causing autosomal recessive, juvenile Parkinsonism (*PINK1*, Parkin, *DJ-1*) (Bonifati et al., 2003; Kitada et al., 1998; Paisán-Ruíz et al., 2004; Polymeropoulos, 1997; Valente et al., 2004; Zimprich et al., 2004). Recently, using an exome sequencing-based approach, 2 independent groups have identified a missense mutation in vacuolar protein sorting 35 homolog (*VPS35* c.1858G>A; p.Asp620Asn) as the probable cause of late-onset PD in a number of kindreds (Vilariño-Güell et al., 2011; Zimprich et al., 2011). Pathogenicity is supported by segregation, evolutionary conservation at that base, and software predictions that the variant is likely to be damaging. However, no other pathogenic mutations have been identified with certainty in *VPS35*, and the c1858G>A mutation, thus far, has only been found in Caucasian individuals. Additional evidence that VPS35 dysfunction predisposes to familial Parkinson's disease will come from identification of other mutations which segregate with Parkinson's disease in families, and the observation that *VPS35* mutations occur in PD patients in other populations.

VPS35 is a highly-conserved component of the retromer, a heteropentameric complex that mediates retrograde transport of transmembrane cargo from endosomes back to the trans-Golgi network (Bonifacino and Rojas, 2006; Seaman, 2005). In mammals, the full structure comprises a sorting nexin dimer, composed of either sorting nexins (SNX) 1 or 2 and 5 or 6, as well as a cargo-recognition trimer, composed of VPS26, VPS29 and VPS35 (Bonifacino and Hurley, 2008). It has been hypothesized that the putative pathogenic mutation in VPS35 may act by disrupting recognition and binding to key cargo proteins (Zimprich et al., 2011). In terms of neurodegenerative disease, recent work showing that VPS35 can associate directly with both soluble N-ethylmaleimide-sensitive factor attachment protein receptor (SNARE) proteins and the sortilin-related receptor SORL1 is thus particularly interesting (Arighi et al., 2004; Bonifacino and Hurley, 2008; Nielsen et al., 2007).

## 2 Methods

To estimate the frequency of *VPS35* mutations and identify novel variants in our cohort of PD cases, we sequenced all 17 exons of *VPS35* in 96 familial PD cases, and exon 15 (in which the c.1858G>A; p.Asp620Asn mutation is found) in an additional 64 familial PD cases, 175 young-onset PD cases, and 262 sporadic, neuropathologically confirmed PD cases (total number screened, 501). Neuropathologically confirmed cases were selected from the Queen Square and (Parkinson's disease United Kingdom) brain bank. The study was approved by the East Central London Research Ethics Committee 1 and informed consent was obtained as per its guidelines.

All living patients fulfilled criteria for clinical diagnosis of PD at the time of the study with at least 2 of 3 cardinal signs of tremor, rigidity, and bradykinesia, as well as a positive response to levodopa therapy. Familial cases were defined as those reporting 1 or more first degree relatives with PD with a pedigree consistent with autosomal dominant inheritance and negative testing for known mutations in *SNCA* and *LRRK2*. Young-onset PD cases were defined as those who had developed their first signs of the disease at age 45 or younger (mean age of onset in young-onset PD was 37 ± 6, range 14–45 with a male to female ratio of 1.63:1). A family history of PD (first or second degree relative) was noted in 31 young-onset PD cases (17.7%). The neuropathological diagnosis for brain bank cases was made by an experienced neuropathologist and based on accepted morphological criteria (Ince et al., 2008). This group included 166 males and 96 females, with an average age at onset of 64 ± 11 years (range, 29–85) and a family history recorded in 7.6% of cases.

Genomic DNA was obtained from peripheral blood lymphocytes for all subjects using standard protocols. *VPS35* exons 2–12 are located within a region that is duplicated 12 megabases (Mb) upstream. Primer pairs were designed specifically to amplify these exons (available on request). Polymerase chain reaction (PCR) products were purified from unincorporated nucleotides using a Millipore PCR purification plate (Millipore, Co.Cork, Ireland). A total volume of 8 μL of the clean product and 10 pM of primer (forward or reverse) was used for sequencing. Amplicons were sequenced using the dideoxynucleotide terminator chemistry and a 3730xl DNA Analyzer (Applied Biosystems, Foster City, CA, USA). Sequences were analyzed using Sequencher software (Gene Codes Corporation, Ann Arbor, MI, USA, version 4.10.1).

## 3 Results

Screening revealed a single case from our familial PD cohort who harbored the previously described p.Asp620Asn mutation. No other nonsynonymous variants were detected in any other exon of any sample. The synonymous coding and noncoding variants detected are summarized in Table 1.

**Fig. 1:**
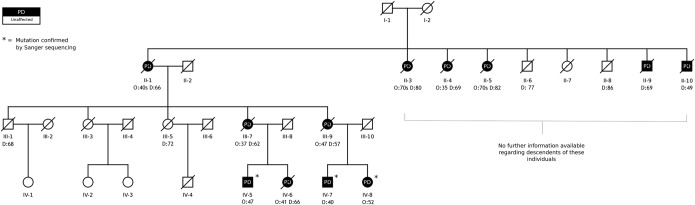
Pedigree of a family showing autosomal dominant inheritance of Parkinson's disease (PD). Age of onset (O:) and age at death (D:) are indicated where known for all descendants of I-1 and I-2. The p.Asp620Asn mutation of *VPS35* was confirmed by Sanger sequencing in the 3 living individuals affected by the disease.

A sibling and first cousin of the case in whom the p.Asp620Asn mutation was identified, both also affected with PD, were also found to carry the same mutation. The family is of European ancestry, the pedigree (Fig. 1) is consistent with autosomal dominant mode of inheritance and revealed 9 further affected individuals across 3 generations, all of whom are deceased.

The index case (IV-7) noticed his first symptoms in 1993 at the age of 40, consisting of stiffness in the left arm on waking followed, a few months later, by gradually worsening stiffness in the left leg. He was diagnosed as suffering from PD in 1995. At that time, examination showed unilateral bradykinesia and reduced arm swing. L-dopa was commenced with a good response, although he continued to gradually worsen. He began to notice a mild intermittent tremor of the left upper limb 13 years after his initial symptoms, followed by the spread of the stiffness to his right upper limb. Dyskinesias started after 9 years of L-dopa therapy, but were not severe. He complains of difficulty with balance, but has not had any falls. There is no history of mood disturbance or other psychiatric features, including visual hallucinations. Cognitively, he feels that his concentration has deteriorated somewhat over the years, but he has only recently retired from a demanding job. A Mini Mental State Examination (MMSE) performed 18 years into the disease revealed a score of 28/30. Olfactory testing was undertaken 18 years into the disease, using the 40-item University of Pennsylvania Smell Identification Test (UPSIT) (Doty et al., 1984) and results were interpreted in comparison with 55 control subjects (27 males and 28 females) and 46 PD patients (30 males and 16 females) who had been previously tested for a study about olfaction in the UK (Silveira-Moriyama et al., 2009). The patient correctly identified 29/40 items of the UPSIT, scoring on the 37th percentile of 55 controls aged between 55 and 65 from the UK, and the 87th percentile of 46 PD subjects aged between 55 and 65 from the UK.

His sibling (IV-8) developed symptoms aged 52 years of age and was formally diagnosed 2 years later. Initial symptoms included cramping in the left foot and tripping when walking. Within 6 months she developed a unilateral leg tremor. Further symptoms included micrographia, and freezing. Dopamine agonists were tried initially but within 3 years she required L-dopa. She has not developed any dyskinesias. She does not report any cognitive symptoms and a Mini Mental State Examination was 30/30. Olfactory testing was undertaken 11 years into the disease as described above with the patient also identifying 29/40 items of the UPSIT. Individual IV-5 presented with a stiffness and rigidity of the left leg at the age, followed by walking difficulties, bradykinesia, loss of postural reflexes and micrographia aged 47. There was no rest tremor. He was diagnosed shortly afterward. He responded well to L-dopa, but developed dyskinesias after 10 to 15 years of treatment and has now had a deep brain stimulator implanted to good effect. There have been no hallucinations or psychiatric complications. The clinical history for individual IV-6 was provided by her brother. She developed PD at the age of 41 with noticeable tremor and generalized bradykinesia and rigidity. She responded well to L-dopa, developing only mild dyskinesias after a reasonable period of treatment. She underwent a pallidotomy at around 60 years of age and died at age 66 as a consequence of a nosocomial infection after a hip replacement operation.

Limited information is available for other affected family members. Individual II-1 presented with akinetic-rigid parkinsonism in her late fourth decade, family members noted that she never developed tremor, but suffered with depression late in the disease course. She died aged 66 years. Individuals II-3 and II-5 developed parkinsonism in their seventh decade and died in their eighth decade. II-4 developed tremor-predominant Parkinson's disease aged 35 years and had a slowly progressive disease course, dying at the age of 69. Individuals II-9 and II-10 are known to have been affected with parkinsonism, but ages of onset are not known. They died in their sixth and fourth decade respectively. Individual III-7 developed parkinsonism at age 37. Medical records and family members indicate left-sided rigidity, bradykinesia, hypomimia, falls, and freezing. Tremor was not recorded. Surgical interventions, including pallidotomy (right then left) and thalamotomy, were performed before L-dopa was commenced. No dyskinesias developed despite 10 years of treatment and she died at age 62. Individual III-9 developed parkinsonism aged 47 years presenting with a unilateral rest tremor. Family members report early severe muscular spasms, difficulty walking, and falls. Later in the disease, she developed depression and cognitive impairment. She died aged 57 years.

## 4 Discussion

Recently, using exome sequencing, 2 groups have independently identified a single c.1858G>A mutation in *VPS35* as the probable cause of Parkinson's disease in several kindreds. Therefore, in this study, we screened our own Parkinson's population for variants in this gene, both in order to estimate the frequency of the published mutations and in order to search for novel mutations that may be disease-causing. We included PD patients with a positive family history, as well as those with early onset and late-onset sporadic disease in order to maximize our chances of identifying mutations.

We identified 1 individual with the published putative disease-causing mutation (c.1858G>A; p.Asp620Asn). This individual had a family history that was consistent with highly penetrant, autosomal dominant Parkinson's disease. Based on clinical examination, notes and family reports, the disease in this family appears clinically similar to idiopathic PD. Onset was generally unilateral, with slow progression and variable tremor. Cognitive or psychiatric features are not prominent. Response to L-dopa was generally good and was not associated with early or unusually severe dyskinesias. The 2 patients in which olfaction was tested here show mild to moderate olfactory dysfunction when compared with controls in the UK, but still performed better than the vast majority of PD patients. Olfaction has been demonstrated to be unimpaired in homozygous and compound heterozygous carriers of Parkin mutations (Alcalay et al., 2011; Khan et al., 2004) but impaired in *LRRK2* parkinsonian carriers (Healy et al., 2008; Saunders-Pullman et al., 2011; Silveira-Moriyama et al., 2008, 2010), although not to the same extent as in sporadic PD. The most notable feature of our kindred is the relatively early age at onset. Six individuals developed PD in their third or fourth decades, of which 4 were younger than the age of 45.

No other potentially disease-causing mutations were found in exon 15 (597 cases screened) or in any other exon (96 cases screened). It would seem reasonable to conclude, therefore, that the recently published *VPS35* c.1858G>A mutation, is not a common cause of PD, in our population.

## Disclosure statement

The authors declare no conflicts of interest.

All DNA samples were obtained in accordance with the approval of local ethics committees. The study was approved by the East Central London Research Ethics Committee 1 and informed consent was obtained as per its guidelines.

## Figures and Tables

**Table 1:** Summary of variants found in *VPS35*

Variant	Nucleotide change	Amino acid change	Rs number (if known)	Exon	Number of cases
Coding					
Nonsynonymous	c.1858G>A	p.Asp620Asn	Recently published	15	1
Synonymous	c.231T>C	p.Leu77Leu	rs11550462	4	1
	c.1842T>C	p.Tyr615Tyr	Novel	15	1
	c.1938C>T	p.His646His	rs168745	15	1
Noncoding					
UTR	c.1-34G>A		rs3743928		1
Intronic	c.3+25A>G		Novel	1	1
	c.1524+42G>C		rs4966616	12	27 (2 homozygotes)
	c.1648-26G>A		rs2304492	14	58 (10 homozygotes)

Key: UTR, (untranslated region).

## References

[bib1] Alcalay R.N., Siderowf A., Ottman R., Caccappolo E., Mejia-Santana H., Tang M.X., Rosado L., Louis E., Ruiz D., Waters C., Fahn S., Cote L., Frucht S., Ford B., Orbe-Reilly M., Ross B., Verbitsky M., Kisselev S., Comella C., Colcher A., Jennings D., Nance M., Bressman S., Scott W.K., Tanner C., Mickel S., Rezak M., Novak K.E., Friedman J.H., Pfeiffer R., Marsh L., Hiner B., Clark L.N., Marder K. (2011). Olfaction in Parkin heterozygotes and compound heterozygotes: the CORE-PD study. Neurology.

[bib2] Arighi C.N., Hartnell L.M., Aguilar R.C., Haft C.R., Bonifacino J.S. (2004). Role of the mammalian retromer in sorting of the cation-independent mannose 6-phosphate receptor. J. Cell Biol.

[bib3] Bonifacino J.S., Hurley J.H. (2008). Retromer. Curr. Opin. Cell Biol.

[bib4] Bonifacino J.S., Rojas R. (2006). Retrograde transport from endosomes to the trans-Golgi network. Nat. Rev. Mol. Cell Biol.

[bib5] Bonifati V., Rizzu P., Van Baren M.J., Schaap O., Breedveld G.J., Krieger E., Dekker M.C., Squitieri F., Ibanez P., Joosse M., Van Dongen J.W., Vanacore N., Van Swieten J.C., Brice A., Meco G., Van Duijn C.M., Oostra B.A., Heutink P. (2003). Mutations in the DJ-1 gene associated with autosomal recessive early-onset parkinsonism. Science.

[bib6] Braak H., Del Tredici K., Rüb U., De Vos R.A., Jansen Steur E.N., Braak E. (2003). Staging of brain pathology related to sporadic Parkinson's disease. Neurobiol. Aging.

[bib7] Doty R.L., Shaman P., Dann M. (1984). Development of the University of Pennsylvania Smell Identification Test: a standardized microencapsulated test of olfactory function. Physiol. Behav.

[bib8] Healy D.G., Falchi M., O'Sullivan S.S., Bonifati V., Durr A., Bressman S., Brice A., Aasly J., Zabetian C.P., Goldwurm S., Ferreira J.J., Tolosa E., Kay D.M., Klein C., Williams D.R., Marras C., Lang A.E., Wszolek Z.K., Berciano J., Schapira A.H.V., Lynch T., Bhatia K.P., Gasser T., Lees A.J., Wood N.W. (2008). International LRRK2 Consortium, Phenotype, genotype, and worldwide genetic penetrance of LRRK2-associated Parkinson's disease: a case-control study. Lancet Neurol.

[bib9] Ince P.G., Clark B., Holton J.L., Revesz T., Wharton S., Love S., Louis D.N., Ellison D.W. (2008). Disorders of movement and system degenerations. Greenfield's Neuropathology.

[bib10] Nalls M.A., Plagnol V., Hernandez D.G., Sharma M., Sheerin U.M., Saad M., Simón-Sánchez J., Schulte C., Lesage S., Sveinbjörnsdóttir S., Stefánsson K., Martinez M., Hardy J., Heutink P., Brice A., Gasser T., Singleton A.B., Wood N.W., International Parkinson Disease Genomics Consortium (2011). Imputation of sequence variants for identification of genetic risks for Parkinson's disease: a meta-analysis of genome-wide association studies. Lancet.

[bib11] International Parkinson's Disease Genomics Consortium (IPDGC), Wellcome Trust Case Control Consortium 2 (WTCCC2) (2011). A Two-Stage Meta-Analysis Identifies Several New Loci for Parkinson's Disease. PLoS Genet.

[bib12] Khan N.L., Katzenschlager R., Watt H., Bhatia K.P., Wood N.W., Quinn N., Lees A.J. (2004). Olfaction differentiates parkin disease from early-onset parkinsonism and Parkinson disease. Neurology.

[bib13] Kitada T., Asakawa S., Hattori N., Matsumine H., Yamamura Y., Minoshima S., Yokochi M., Mizuno Y., Shimizu N. (1998). Mutations in the parkin gene cause autosomal recessive juvenile parkinsonism. Nature.

[bib14] Mayeux R., Marder K., Cote L.J., Denaro J., Hemenegildo N., Mejia H., Tang M.X., Lantigua R., Wilder D., Gurland B. (1995). The frequency of idiopathic Parkinson's disease by age, ethnic group, and sex in northern Manhattan, 1988–1993. Am. J. Epidemiol.

[bib15] Nielsen M.S., Gustafsen C., Madsen P., Nyengaard J.R., Hermey G., Bakke O., Mari M., Schu P., Pohlmann R., Dennes A., Petersen C.M. (2007). Sorting by the cytoplasmic domain of the amyloid precursor protein binding receptor SorLA. Mol. Cell. Biol.

[bib16] Paisán-Ruíz C., Jain S., Evans E.W., Gilks W.P., Simón J., Van Der Brug M., López De Munain A., Aparicio S., Gil A.M., Khan N., Johnson J., Martinez J.R., Nicholl D., Carrera I.M., Pena A.S., De Silva R., Lees A., Martí-Massó J.F., Pérez-Tur J., Wood N.W., Singleton A.B. (2004). Cloning of the gene containing mutations that cause PARK8-linked Parkinson's disease. Neuron.

[bib17] Poewe W., Mahlknecht P. (2009). The clinical progression of Parkinson's disease. Parkinsonism Relat. Disord.

[bib18] Polymeropoulos M.H., Lavedan C., Leroy E., Ide S.E., Dehejia A., Dutra A., Pike B., Root H., Rubenstein J., Boyer R., Stenroos E.S., Chandrasekharappa S., Athanassiadou A., Papapetropoulos T., Johnson W.G., Lazzarini A.M., Duvoisin R.C., Di Iorio G., Golbe L.I., Nussbaum R.L. (1997). Mutation in the alpha-synuclein gene identified in families with Parkinson's disease. Science.

[bib19] Saunders-Pullman R., Stanley K., Wang C., San Luciano M., Shanker V., Hunt A., Severt L., Raymond D., Ozelius L.J., Lipton R.B., Bressman S.B. (2011). Olfactory dysfunction in LRRK2 G2019S mutation carriers. Neurology.

[bib20] Seaman M.N. (2005). Recycle your receptors with retromer. Trends Cell Biol.

[bib21] Silveira-Moriyama L., Guedes L.C., Kingsbury A., Ayling H., Shaw K., Barbosa E.R., Bonifati V., Quinn N.P., Abou-Sleiman P., Wood N.W., Petrie A., Sampaio C., Ferreira J.J., Holton J., Revesz T., Lees A.J. (2008). Hyposmia in G2019S LRRK2-related parkinsonism: clinical and pathologic data. Neurology.

[bib22] Silveira-Moriyama L., Munhoz R.P., De J Carvalho M., Raskin S., Rogaeva E., De C Aguiar P., Bressan R.A., Felicio A.C., Barsottini O.G.P., Andrade L.A., Chien H.F., Bonifati V., Barbosa E.R., Teive H.A., Lees A.J. (2010). Olfactory heterogeneity in LRRK2 related Parkinsonism. Mov. Disord.

[bib23] Silveira-Moriyama L., Petrie A., Williams D.R., Evans A., Katzenschlager R., Barbosa E.R., Lees A.J. (2009). The use of a color coded probability scale to interpret smell tests in suspected parkinsonism. Mov. Disord.

[bib24] Valente E.M., Abou-Sleiman P.M., Caputo V., Muqit M.M., Harvey K., Gispert S., Ali Z., Del Turco D., Bentivoglio A.R., Healy D.G., Albanese A., Nussbaum R., González-Maldonado R., Deller T., Salvi S., Cortelli P., Gilks W.P., Latchman D.S., Harvey R.J., Dallapiccola B., Auburger G., Wood N.W. (2004). Hereditary early-onset Parkinson's disease caused by mutations in PINK1. Science.

[bib25] Vilariño-Güell C., Wider C., Ross O.A., Dachsel J.C., Kachergus J.M., Lincoln S.J., Soto-Ortolaza A.I., Cobb S.A., Wilhoite G.J., Bacon J.A., Behrouz B., Melrose H.L., Hentati E., Puschmann A., Evans D.M., Conibear E., Wasserman W.W., Aasly J.O., Burkhard P.R., Djaldetti R., Ghika J., Hentati F., Krygowska-Wajs A., Lynch T., Melamed E., Rajput A.H., Rajput A.H., Solida A., Wu R.-M., Uitti R.J., Wszolek Z.K., Vingerhoets F., Farrer M.J. (2011). VPS35 Mutations in Parkinson Disease. Am. J. Hum. Genet.

[bib26] Zimprich A., Benet-Pagès A., Struhal W., Graf E., Eck S.H., Offman M.N., Haubenberger D., Spielberger S., Schulte E.C., Lichtner P., Rossle S.C., Klopp N., Wolf E., Seppi K., Pirker W., Presslauer S., Mollenhauer B., Katzenschlager R., Foki T., Hotzy C., Reinthaler E., Harutyunyan A., Kralovics R., Peters A., Zimprich F., Brücke T., Poewe W., Auff E., Trenkwalder C., Rost B., Ransmayr G., Winkelmann J., Meitinger T., Strom T.M. (2011). A mutation in VPS35, encoding a subunit of the retromer complex, causes late-onset Parkinson disease. Am. J. Hum. Genet.

[bib27] Zimprich A., Biskup S., Leitner P., Lichtner P., Farrer M., Lincoln S., Kachergus J., Hulihan M., Uitti R.J., Calne D.B., Stoessl A.J., Pfeiffer R.F., Patenge N., Carbajal I.C., Vieregge P., Asmus F., Müller-Myhsok B., Dickson D.W., Meitinger T., Strom T.M., Wszolek Z.K., Gasser T. (2004). Mutations in LRRK2 cause autosomal-dominant parkinsonism with pleomorphic pathology. Neuron.

